# Characterization and performance analysis of composite bioplastics synthesized using titanium dioxide nanoparticles with corn starch

**DOI:** 10.1016/j.heliyon.2019.e02009

**Published:** 2019-08-28

**Authors:** Md. Ruhul Amin, Mohammad Asaduzzaman Chowdhury, Md. Arefin Kowser

**Affiliations:** Department of Mechanical Engineering, Dhaka University of Engineering and Technology, Gazipur, 1707, Bangladesh

**Keywords:** Materials science, Materials chemistry, Starch, Bioplastic, Composite bioplastic, Characterization

## Abstract

Plastic is an amazing material, and wonderful invention, it has changed the world. Plastic is used everywhere and every day across the globe. But despite its varied uses, its disposal has threatened the environment. Biodegradable plastics can meet these needs and can easily be disposed to the environment. This work focuses on the characterization and performance analysis of starch bioplastics and composite bioplastic to reduce the plastic pollution by its various uses. TGA, DSC, SEM, FTIR, and surface roughness analyses were used to characterize, the mechanical properties, thermal properties and the morphology of the starch bioplastics and composite bioplastic. Starch bioplastics were fabricated using starch vinegar and glycerol. Composite bioplastics ware fabricated using starch, vinegar, glycerol and titanium dioxide. The addition of titanium dioxide improved the tensile strength of the bioplastics from 3.55 to 3.95 MPa and decreased elongation from 88% to 62%. According to Differential Scanning Calorimetry (DSC) Test, the melting point (T_m_) and Glass Transition Temperature (T_g_) significantly affected by the presence of titanium dioxide (TiO_2_). The degree of nano-composite crystallinity was formed by the strong interfacial interaction between the titanium dioxide nanoparticles and the amorphous region of the chain. The decomposition temperature of starch bioplastic was increased by mixing with titanium dioxide nanoparticles. The results gained from SEM showed that better compatible morphologies in composite bioplastic compared to starch bioplastic for its fewer voids, holes, and crack. The functional group O–H, C–H, C=O, and C–O indicate the formation of starch bioplastics and composite bioplastics has already occurred which was confirmed by FTIR spectroscopy. The result is also verified with the available results of other researchers. Therefore, composite bioplastic is a modified elevation of a starch bioplastic with a modified upgrade feature. It can be an alternative to existing conventional plastic, especially packaging applications.

## 1 Introduction

Plastics are used worldwide from drinking cups to various parts of automobiles and motorbikes. They are imperative to the trade market as well as the packaging of materials all over the world. However, they have been an environmental concern because of its prolonged rate of degradation. Starch is a promising candidate for developing sustainable materials are considered which mainly due to its biodegradable properties, low cost and renewability [1, 2, 3, 4]. A lot of research has been done to develop starch-based polymers to conserve petrochemical resources and reduce environmental impact [1, 2, 3, 4, 5, 6, 7, 8, 9]. However, starch-based materials contain some drawbacks, including long term stability, aging, and poor mechanical properties [6]. A plasticizer such as glycerin has been added to improve shelf-life, elasticity, and limitations of the product [7]. In the presence of the plasticizer, space also occupying between the starch polymer chains, which reduces the plastic crystallinity [8]. This plasticized starch is more versatile and can be blended with various polymeric materials for numerous applications. Various physical or chemical modifications like derivation, graft copolymerization, and blending have been investigated to improve the characteristics of starch bioplastic. The most effective method to increase preference is filler. Cost-effective reinforcements are kenaf [9], Paper pulp [10, 11] pineapple [12], bamboo [13], short abaca [14], flax [15, 16, 17], sisal [18], lyocell [19], jute [20], Cordenka [21], organic renewable resources [22], and microcrystalline cellulose [23]. Composite materials containing nanoparticles and polymers can deliver high-performance innovative materials, and nanofillers have exceptional interfacial interactions in polymer and significantly improve polymer properties [24]. All mechanical and thermal properties of the Biopolymer nanocomposites improved with addition of the bio-fillers to neat polymers [25]. The tensile strength and the Young's modulus increased and the elongation decreased with the increase of the electrochemical-mechanical liquid exfoliation (EMLE) graphene content in the composites [26]. The titanium dioxide nanoparticle enhanced the functional properties of potato starch film [27]. A lot of studies to increase the performance of starch-based bioplastics using fillers with several types of starch such as potato starch [28], wheat starch [29, 30, 31], jackfruit seed starch [32], corn starch [30, 33, 34, 35] and cassava starch [36], have been researched. But corn starch based nanocomposite bioplastic using TiO_2_ nanoparticles have not been investigated so far. So, it would be a new addition to research. The main objectives of this research were the preparation and characterization of composite bioplastics using titanium dioxide nanoparticle with corn starch. The analysis was done to identify the area of application of these materials and to evaluate the potentiality. This research database can be useful for designing and manufacturing biodegradable materials.

## 2 Materials and methods

### 2.1 Materials

Corn starch (20% amylose, 10% moisture, mean particle size 18μm), glycerol, white vinegar and Titanium dioxide nanoparticles (21nm) ware locally purchased from Dhaka (Dhaka, Bangladesh). All other reagents were analytical grade. The following amounts of each ingredient are needed to fabricate the bioplastic (Table 1) (see Tables 2, 3, 4).

**Table 1:** Amount of ingredients for bioplastic making.

Starch bioplastic	Composite bioplastic
• 300 ml distilled water (74%) • 25 gm. glycerol (6%) • 50 gm. corn starch (12%) • 30 ml of white vinegar (8%)	• 300 ml distilled water (69%) • 25 gm. glycerol (5.5%) • 50 gm. corn starch (11.5%) • 30 ml of white vinegar (7%) • 30 gm. Titanium dioxide (7%)

**Table 2:** The main FTIR absorption peaks for starch and composite bioplastic.

Functional Group	Wave number [Cm^−1^ ]	
	Starch Bioplastic	Composite Bioplastic
O–H	3277.76	3270.17
C–H	2926.31	2932.52
C = O	1643.22	1638.66
C–O	1014.48	1019.63

**Table 3:** The decomposition temperature of starch bioplastic and composite bioplastic.

	First step	2nd step
Starch Bioplastic	57–201 °C	220–385 °C
Composite Bioplastic	50–210 °C	240–410 °C

**Table 4:** Glass transition temperature (Tg) and melting temperature (Tm) of starch and composite bioplastic.

	Starch Bioplastic	Composite Bioplastic
Melting Temperature (T_m_)	297 °C	303 °C
Glass Transition Temperature (T_g_)	57.2 °C	66.8 °C

### 2.2 Require tools

**Table:** 

1.	Hot-plate-magnetic-stirrer	01 Nos.
2.	Beake	02 Nos.
3.	Glass tube	01 Nos.
4.	Weight scale	01 Nos
5.	Infrared thermometer gun	01 Nos
6.	Aluminum foil	01Roll

### 2.3 Fabrication process

**Fig. 1:**
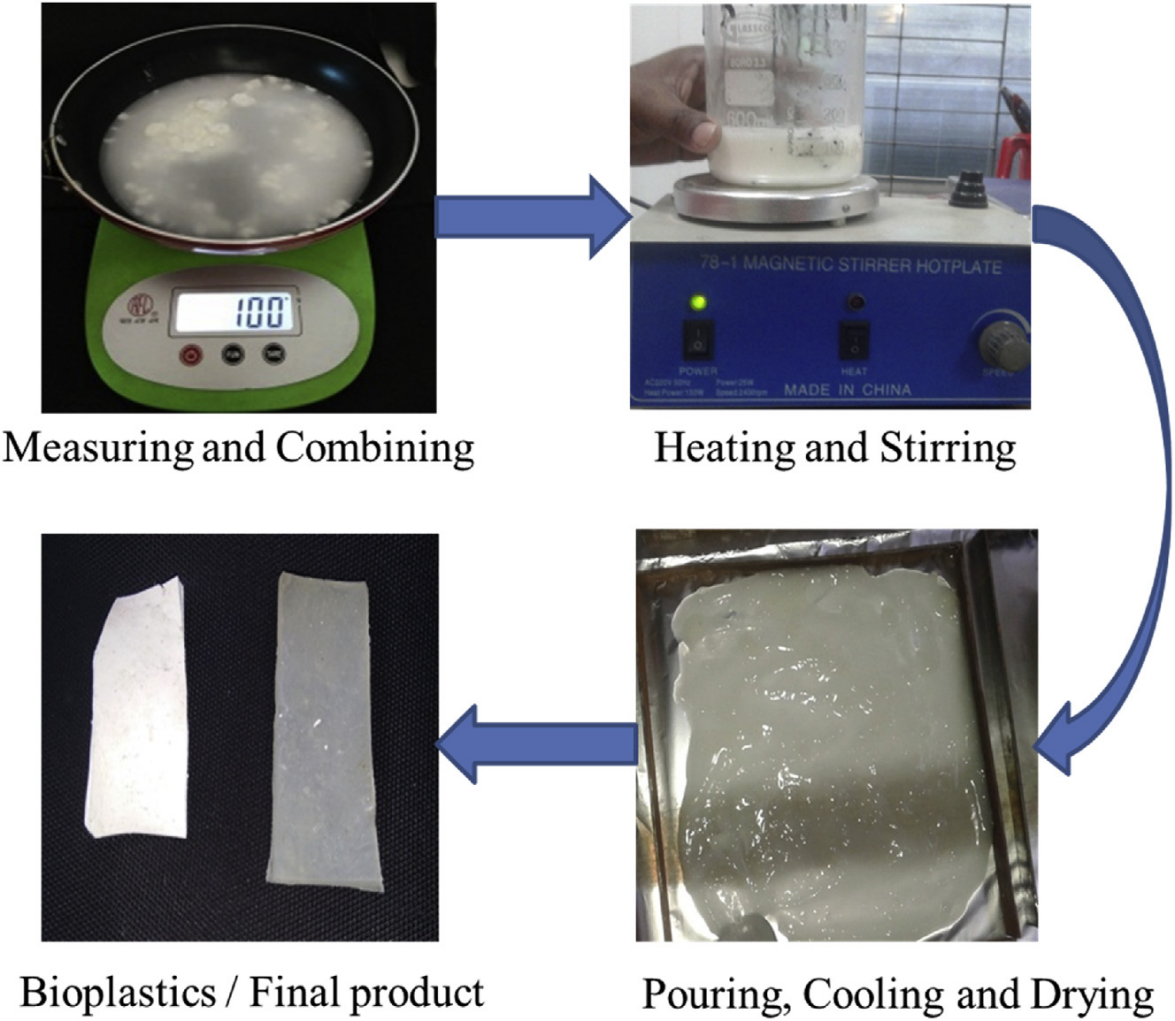
Fabrication process of bioplastics.

To fabricate the starch bioplastic, 50gm of corn starch was added into a beaker together with 300ml of distilled water. Next, 25gm. glycerol and 30 ml of white vinegar were added into the beaker. Then the beaker is placed on the" Hot-plate-magnetic-stirrer" and the mixture is stirred for 5 min. During heating, stirrer should be turned on. This process occurs quickly, so the mixture must be stirring until thickens. Once the mixture is completely ready, it will have to be stir several times and then the mixture should be poured into aluminum foil or dye. Depending on the thickness of the plastic it requires time may be less or more during drying. Then the plastic will be kept in a cool, dry place. After 10 days, bioplastic in sheet form was obtained as shown in Fig. 1. Composite bioplastic made in the same process has just been added extra 30gm. titanium dioxide. However, the mention is that composite bioplastic is not transparent.

## 3 Analysis

### 3.1 Characterization of starch bioplastic and composites bioplastics

#### 3.1.1 Thermal properties

DSC analysis coupled with TGA performed in TA-Instrument SDT650. The weight of the specimen was in the range of 10–25mg and the temperature increased by 5 °C per minute starting from 30 °C up to 500 °C.

#### 3.1.2 SEM analysis

The microstructural analysis of the bioplastics was carried out using a Scanning Electron Microscope (Hitachi S-4800). Two different samples, 0.5 cm^2^ in size, of each bioplastic were fractured after immersion in liquid nitrogen and randomly broken to investigate the surface of the samples. Cryo-fractured samples were mounted on aluminum stubs and fixed on the support using double-sided adhesive tape. Finally, samples were gold palladium coated and observed using an accelerating voltage of 10 kV and a working distance of 10 mm.

#### 3.1.3 FTIR analysis

The FTIR measures were carried out on a Perkin Elmer Spectrum One spectrometer coupled to an Auto Image light microscope. These analyses were performed on samples of starch bioplastic and composites bioplastics.

#### 3.1.4 Mechanical properties

Tensile tests were performed using the CMT-10 Computer Control Electronic Universal Testing Machine. Tensile strength (TS) and elongation were determined from the stress-strain curves, estimated from force-distance data obtained for the different and a strain rate of 2 mm/min at room temperature. All mechanical testing of bioplastics was conditional according to the standard method of ASTM-D638-14. The sample Width of narrow section was 13mm, length of narrow section 57mm, overall length 165mm, thickness 3.5mm and gage length 50mm. There are three specimens ware tested for each sample.

#### 3.1.5 Surface roughness analysis

Surtronic S128 surface roughness tester is used to analyze the surface roughness parameters of starch bioplastic and composite bioplastic.

#### 3.1.6 Soil burial biodegradation test

This analysis was carried out according to the methodology reported by M. Maiti et al. [41], with slight modifications. Starch bioplastic and TiO_2_ composite bioplastic of dimensions 50 mm × 30 mm × 3 mm were placed in the soil at a depth of 10cm. The soil was placed in the laboratory, and the moisture of the soil was maintained by sprinkling water at regular time intervals. The degradation rate of the soil burial test was calculated from weight loss of the sample over time. The biodegradation weight loss was determined for seven days interval by using the following equation-

Weight loss % = (M_i_ −M_f_)/M_i_ × 100 %

Where M_i_ is the initial mass and M_f_ is the final mass of the sample after drying.

## 4 Results & discussion

### 4.1 SEM analysis

**Fig. 2:**
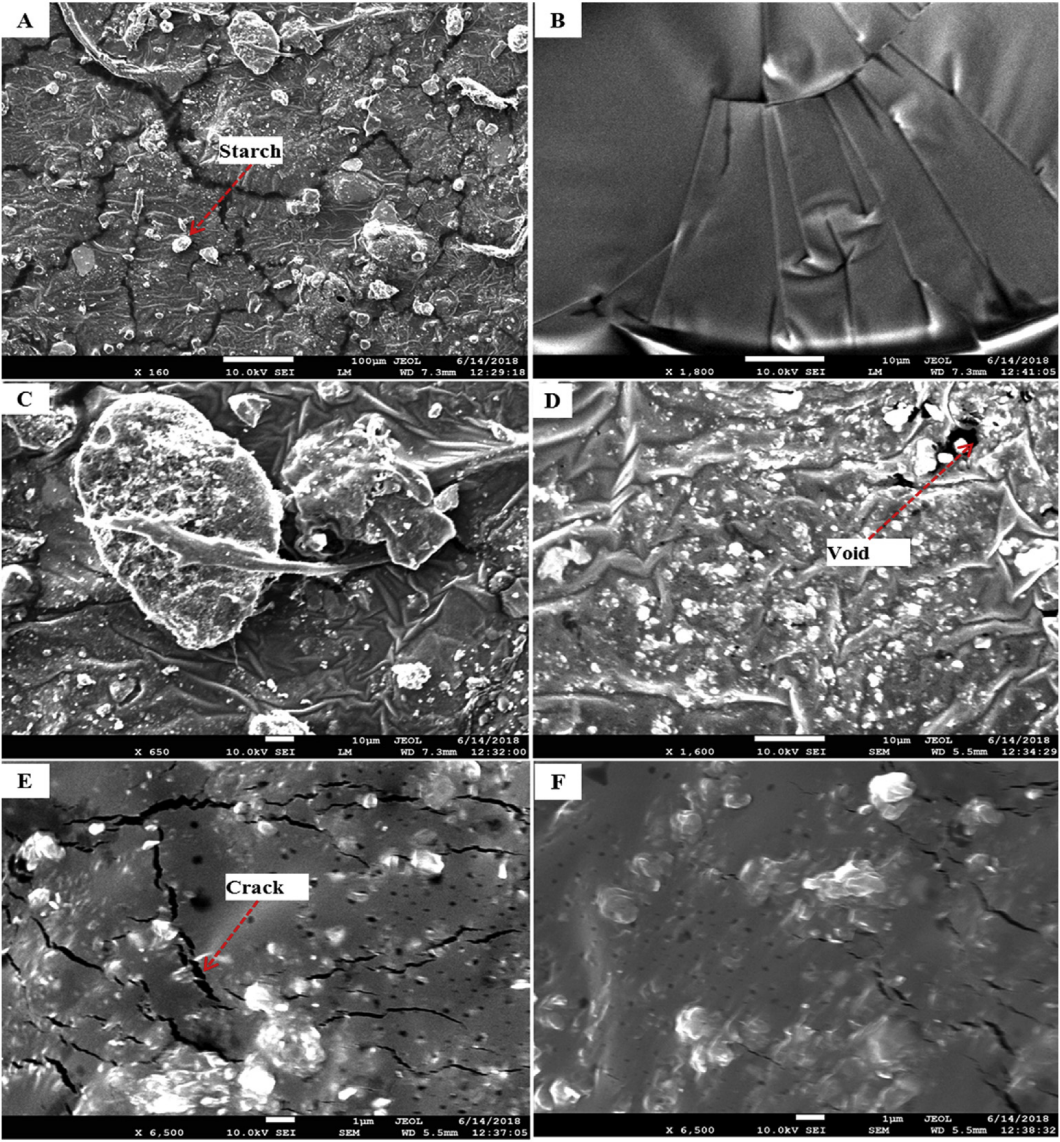
SEM photograph of starch bioplastic (A) Starch bioplastic (160 X), (B) Starch bioplastic (1800 X), (C) Presence of granules (650 X), (D) Presence of void (1600 X), (E)–(F) Presence of crack (6500 X).

**Fig. 3:**
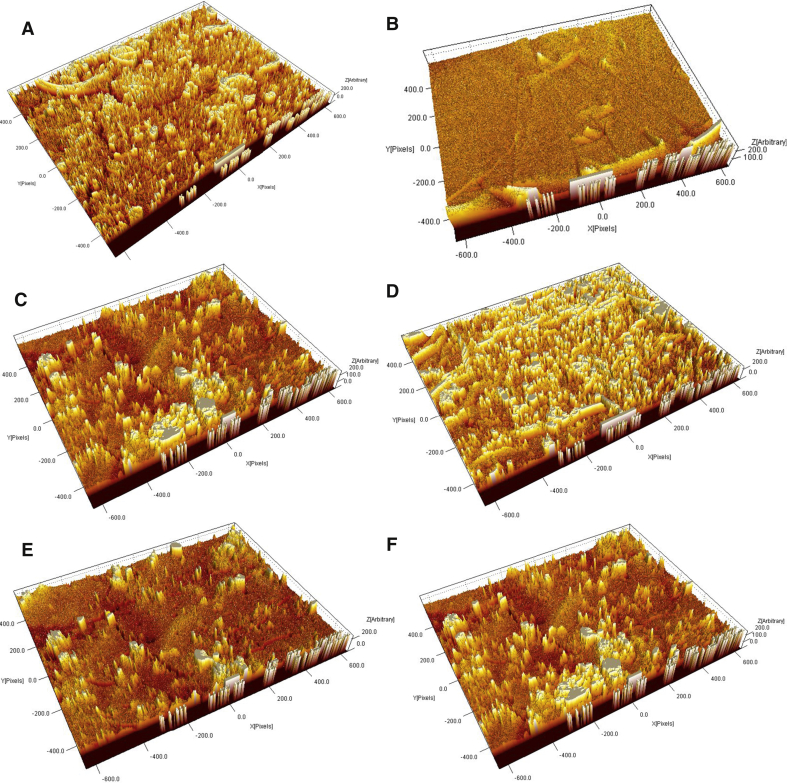
3D AFM micrographs with RMS roughness of starch bioplastic (A) Starch bioplastic (160 X), (B) Starch Bioplastic (1800 X), (C) Presence of granules (650 X), (D) Presence of void (1600 X), (E)–(F) Presence of crack (6500 X).

Fig. 2 and Fig. 3 show surface microstructure and 3D AFM micrographs with RMS roughness of the starch bioplastic. Surface features consist of granules (the remaining part of the starch particle), which means that the starch was not fully gelatinized during the formation process. This outcome is similar to the reports of Hern'andez et al. [37] it has been described that SEM images depicted contain insoluble remnants (i.e. ghosts) from starch granule swelling. Some voids were also visible (black dots are visualized) on the fractured surface that had contributed to the low impact and tensile strength.

**Fig. 4:**
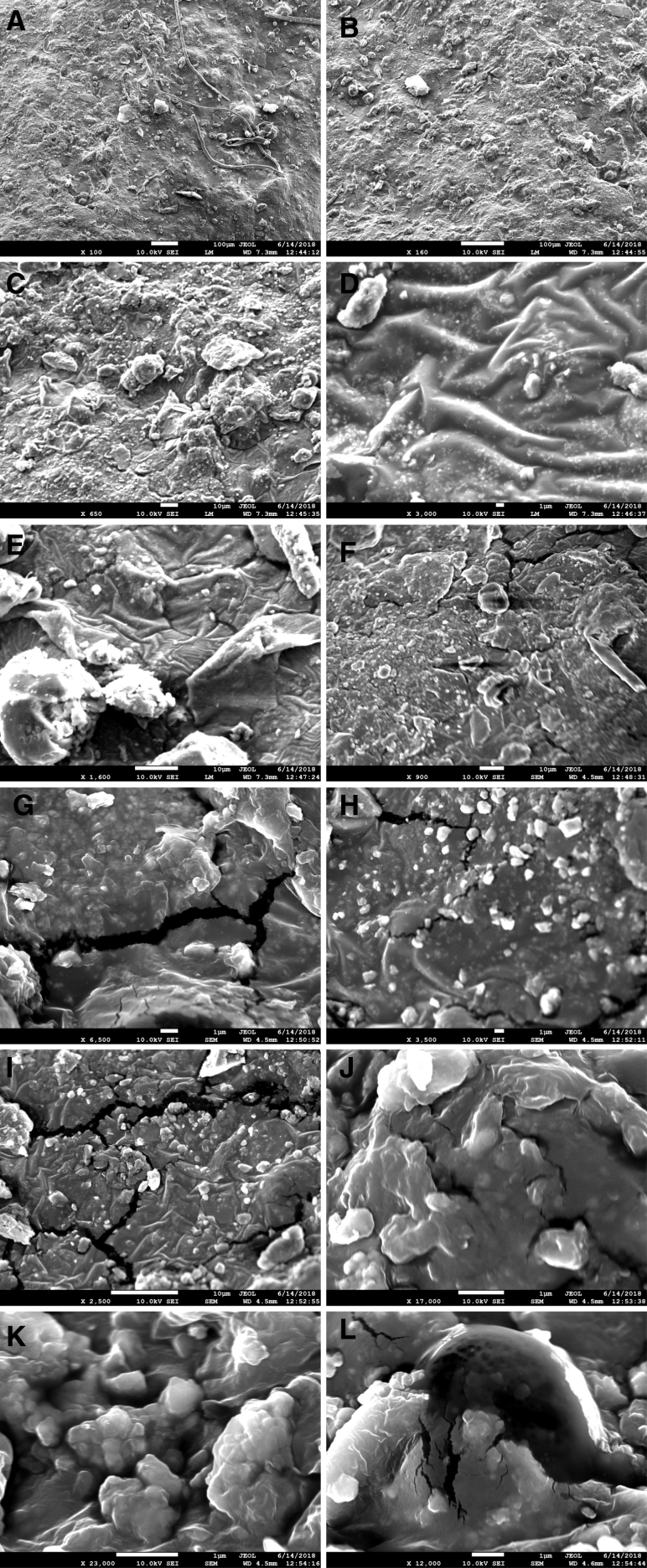
The SEM photograph of composite bioplastic (A) Composite bioplastic (100 X), (B) Composite bioplastic (160 X), (C) Presence of granules (650 X), (D) Presence of non-gelatinized TiO2 granules (3000 X), (E) Composite bioplastic (1600 X), (F) Presence of edge (900 X), (G)–(I) Presence of crack and grooves, (J)–(L) Rough surface with some grooves.

**Fig. 5:**
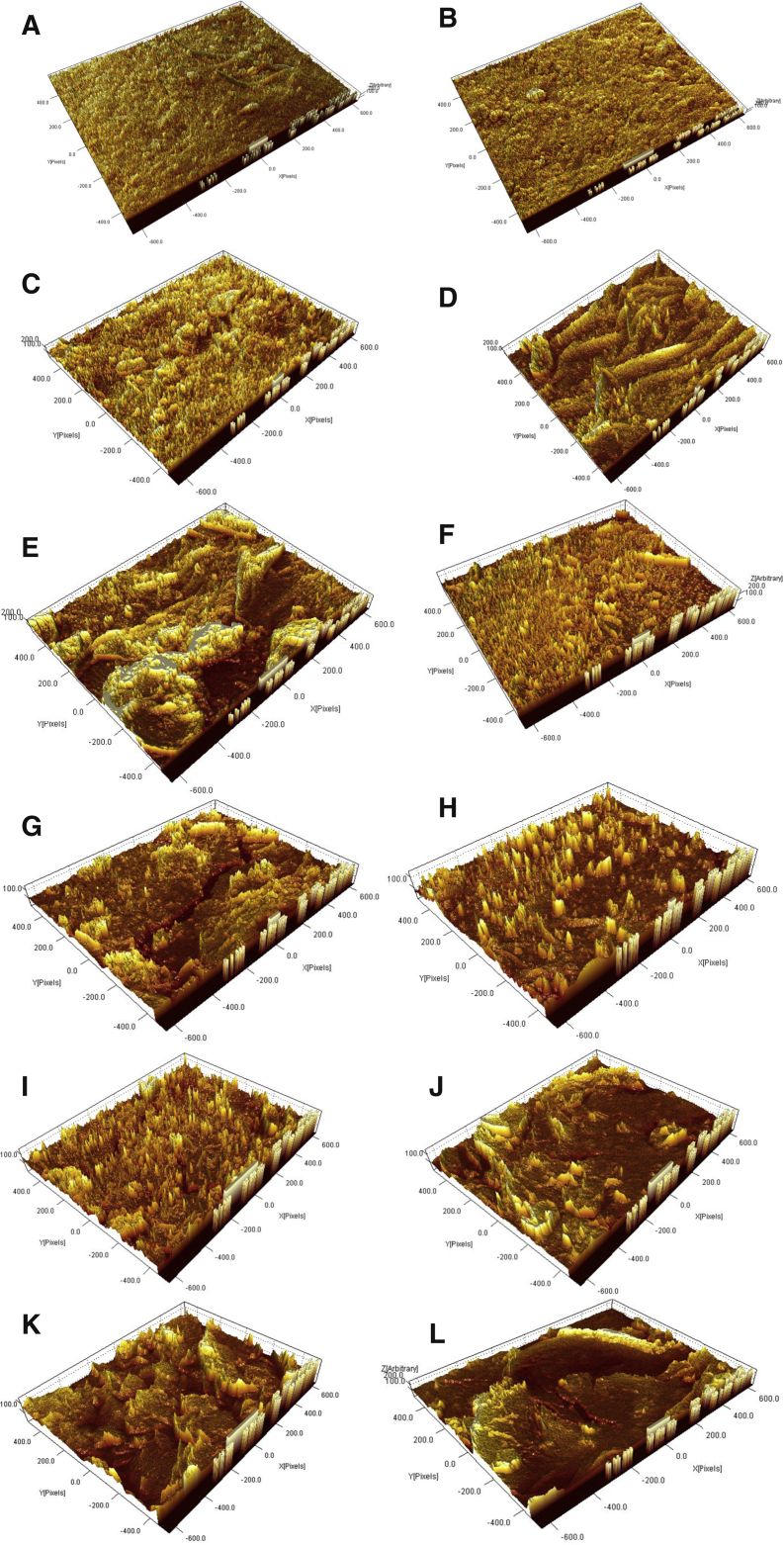
3D AFM micrographs with RMS roughness of composite bioplastic(A) Composite bioplastic (100 X), (B) Composite bioplastic (160 X), (C) Presence of granules (650 X), (D) Presence of non-gelatinized TiO2 granules (3000 X), (E) Composite bioplastic (1600 X), (F) Presence of edge (900 X), (G)–(I) Presence of crack and grooves, (J)–(L) Rough surface with some grooves.

The surface microstructure of composites bioplastics was studied with SEM and 3D AFM micrographs with RMS roughness (Fig. 4 and Fig. 5). Analyses of composite bioplastics surface reveal that the composite bioplastics have an irregular structure, with ridges and grooves. SEM images show that the composite bioplastics surfaces exposed to air are rough with some grooves and presence of non-gelatinized TiO_2_ granules. This morphology is typical of incompatible blends, resulting in a poor tensile property. A similar finding was offered by Selene Harunsyah et al. [42] stated that the morphology structure of cassava starch-clay nanoparticles and plasticizer glycerin bioplastic has not given homogeneous morphology structure. Few voids, edge, holes and poor interfacial adhesion observed in the morphologies of the surface. Few crack propagations in the composite bio-plastics indicates poor bonding between the components.

The starch bioplastic presented a uniform and smoother surface than starch composite bioplastic. The presence of the remaining part of the starch particle and non-gelatinized TiO_2_ granules is higher in composite bioplastic than starch bioplastic. The surface roughness starch bioplastic increased and the uniformity decreased by the TiO_2_ nanoparticles. Fewer voids, holes, and crack shows better compatible morphologies in starch composite bioplastic compared to starch bioplastic.

### 4.2 FTIR analysis

**Fig. 6:**
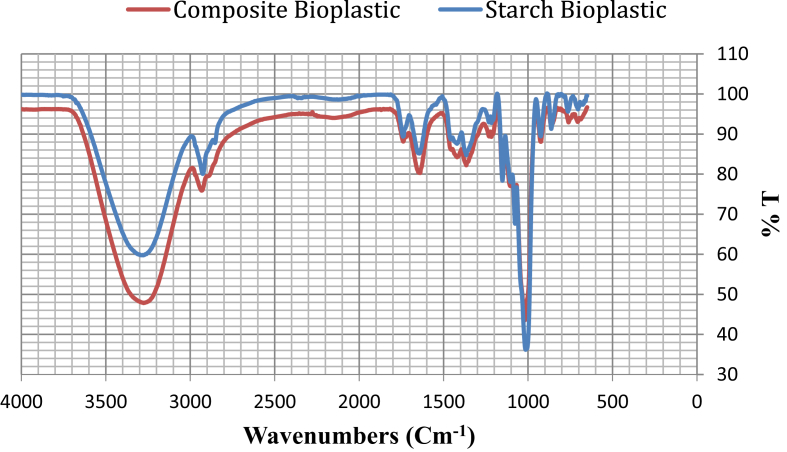
FTIR of starch bioplastic and composite bioplastic.

The FTIR spectra of corn starch bioplastic are shown in Fig. 6. In the spectrum for starch bioplastic, the broadband at 3277.76 cm^−1^ was the OH stretching. A small peak near 1643.22 cm^−1^ was due to the C=O stretching and a peak at 1740 cm^−1^ suggested the presence of a carbonyl group in the film. The peak at 2926.31 cm^−1^ corresponded to the C–H stretching. The bands from 704.27 to 1014.48 cm^−1^ corresponded to the C–O bond stretching.

In the FTIR spectrum of composite bioplastic, the absorption peaks at 3270.17 cm^−1^ is determined to stretching vibrational band of free –OH groups. It indicates an increase in the number of hydrogen bonds between TiO_2_ and hydroxyl groups of the plastics components. The other probable reason was the electrostatic interactions (O⋯Ti) between –OH groups of starch and Ti^2+^ atoms. The absorption band of the C–H and C–O–H bending are shown at the wavenumbers 2932.52 and 1152.92 cm^−1^ respectively, Similarly, Seyed Amir et al. [27] stated that the difference in the absorption peak intensity of C–H and C–O–H at the wavenumbers 2931 and 1154 cm^−1^, it is confirming the possible electrostatic interaction between the starch chains and titanium dioxide.

### 4.3 Thermo gravimetric analysis (TGA)

**Fig. 7:**
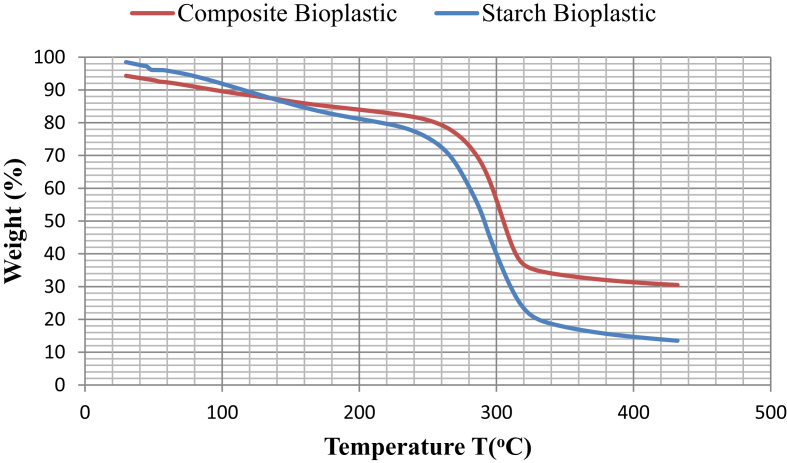
TGA curves for starch bioplastic and composite bioplastic.

Thermogravimetric analysis (TGA) of starch bioplastic and composite bioplastic decomposition graphs is shown in Fig. 7. Starch bioplastic has a 2-step process mechanism of decompositions. In the first step, the moisture of starch bioplastic is evaporated at 57–201 °C. In this stage, the very light volatile matter compounds (vinegar) are lost, and the thermal decomposition process occurs due to evaporation of the water. The thermal decomposition of starch bioplastic occurred in the second stage between 220-385 °C. In the graph, the mass of glycerol starts to evaporate at 220 °C and entirely evaporate at 350 °C.

TGA of the bioplastic composite shows two-step degradation. The first step is attributed to the evaporation of moisture contained in the starch-based bioplastic composite and the second stage between 240-410 °C indicate thermal decomposition of composite bioplastic TiO_2_ is fully decomposed at 410 °C.

The decomposition graphs of starch bioplastic and composite bioplastic are shown the 50% of weight loss occurred at 291 °C and 303 °C, respectively for starch bioplastic and composite bioplastics. Nurul et al. [28] also mentioned in their study that the T_50%_ (temperature at which 50% of weight loss occurred) are at 250 °C and 310 °C, respectively for yam and potato bioplastics. The overall results of the thermal and physical properties analysis of the bio-plastic can be concluded that the major loss in the weight is 4–70% with titanium dioxide reinforcement and 2–87% without TiO_2_ within the range of 30 °C–500 °C. The decomposition temperature of composite bioplastic is higher than starch bioplastic. It can be concluded that starch bioplastic increases the decomposition temperature when adding TiO_2_ nanoparticles as well as indicates that composite bioplastic has greater heat stability compared to starch bioplastic.

### 4.4 Differential Scanning Calorimetry (DSC) analysis

**Fig. 8:**
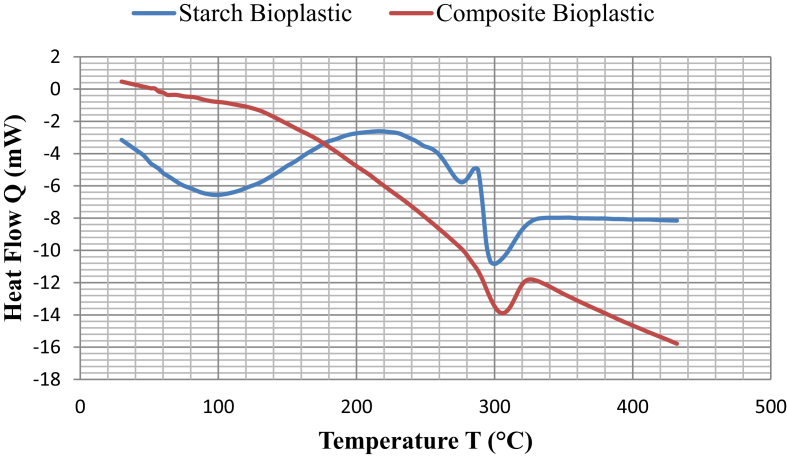
DSC thermograms of starch bioplastic and composite bioplastic.

The thermal properties like melting temperature (T_m_) and glass transition temperature (T_g_) of starch bioplastic and bioplastic composite were studied using DSC as shown in Fig. 8. The maximum melting temperature was discovered at 297 °C for starch bioplastic and 303 °C for composite bioplastic. Starch Bio Plastic has semi-crystal structure, and the crystallization temperature showed at 216 °C. In the DSC thermogram of Starch Bioplastic, there are 2 peaks present between 250 °C and 330 °C. These peaks have been imposed to melting of crystallized amylopectin and co-crystallized amylose. The starch bioplastic and bioplastic composite have only one glass transition temperature (T_g_) which obtained from the results of DSC measurements. The composite bioplastic had a T_g_ of approximately 66.8 °C which was higher T_g_ than starch bioplastic (57.2 °C).

The higher the T_g_ of a polymer, the greater its barrier properties. Because of the glass transit temperature (T_g_) indicates the consistency and miscibility of the components with biopolymer. Identifying only one T_g_ for all the plastic means that all substances contributing to the structure of the bioplastic have been very well spread and consistent with each other. Titanium dioxide nanoparticles were able to form chemical bonds on hydroxyl group sites of the starch chains. These strong bonds can obstruct the motion of the polymer chains and the addition of anti-plasticizers into the starch bioplastic increase the T_g_. TiO_2_ acts as an anti-plasticizer, TiO_2_ significantly shifted T_g_ of composite bioplastic to higher temperatures. Similar to T_g_, the melting point (T_m_) of the composite bioplastic was significantly affected by the presence of TiO_2_ and its values increased when the bioplastic was reinforced with TiO_2_. According to Seyed et al. [27] the melting point of the films has increased dramatically by the presence of titanium dioxide. The reason for this increase is the result of an increase in the degree of nanocomposite crystallinity, which was established by the strong interfacial interaction between the TiO_2_ nanoparticles and the amorphous region of the chain.

### 4.5 Mechanical properties

**Fig. 9:**
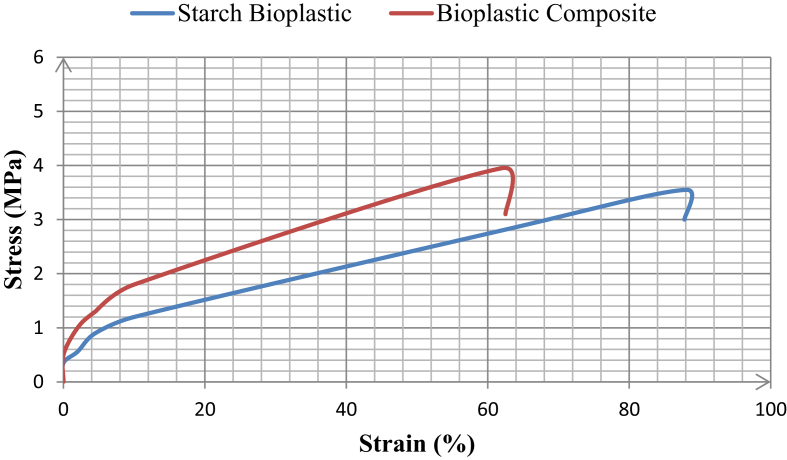
Stress vs. strain curve for starch bioplastic and composite bioplastic.

Fig. 9 and Table 5 show the mechanical properties of Starch bioplastic and composite bioplastic. The results of composite bioplastics have greater mechanical strength than starch-bioplastics but less flexibility. The elongation of starch bioplastic is decreased from 88 to 62 when it changed at composite bioplastic by TiO_2_ nanoparticles. The reason for this behavior can be anti-plasticization. Similar findings were reported by Seyed Amir et al. [27] for the starch bioplastic and composites bioplastics tensile strength 2.66 MPa and 3.86 MPa and elongation 86.70% and 68.43% respectively. By increasing interactions, reducing the free volume between the biopolymer chains with decreasing the flexibility of the bioplastic, titanium dioxide nanoparticles act as an anti-plasticizer.

**Table 5:** Mechanical properties of starch bioplastic and composite bioplastic.

	Tensile strength (MPa)	Elongation (%)
Starch Bioplastic	3.55 ± 0.035	88.1
Composite Bioplastic	3.95 ± 0.039	62.5

### 4.6 Surface roughness analysis

**Fig. 10:**
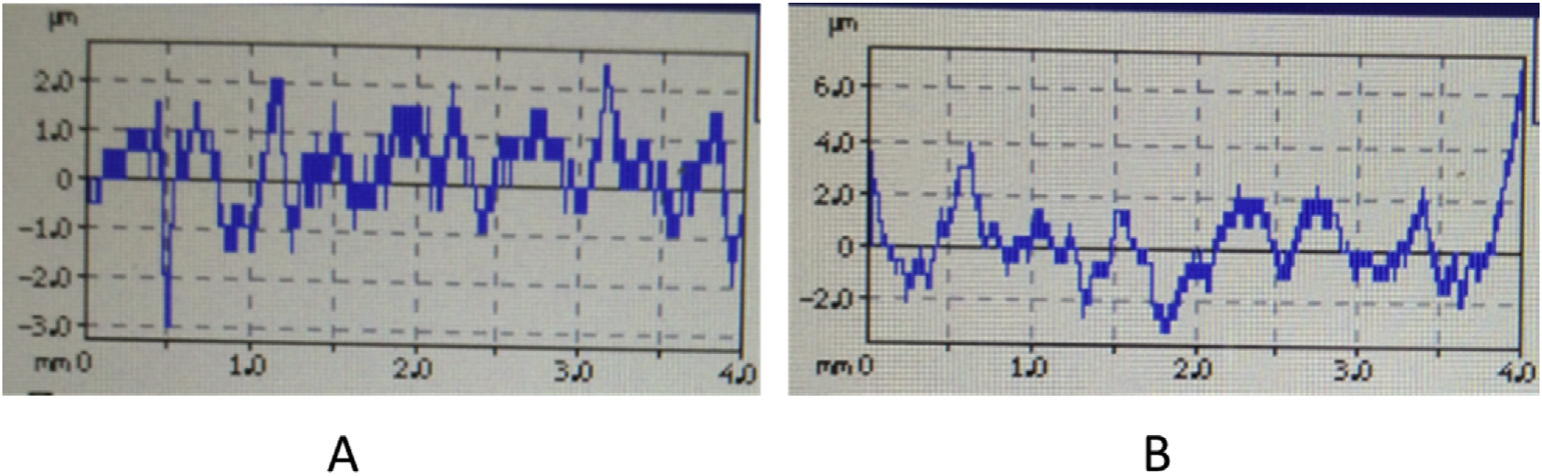
The surface roughness of (A) Starch bioplastic (B) composite bioplastic.

Surtronic S128 surface roughness tester is used to analyze the surface roughness parameters of starch bioplastic and composite bioplastic. Fig. 10 Shown the surface roughness starch bioplastic and composite bioplastic Overall roughness parameters of starch bioplastic (Fig. 10(A)) was estimated to be 0.6 μm (average roughness). The maximum height of roughness (R_t_), maximum peak height (R_p_) and maximum valley depth (R_v_) of starch bioplastic was found to be 5.50 μm, 1.5 μm, and 2.0 μm, respectively. Average roughness, Maximum height of roughness (R_t_), maximum valley depth (R_v_) and maximum peak height (R_p_) of composite bioplastic were found to be 1.0 μm, 10 μm, 2.0 μm, and 3.5 μm, respectively (Fig. 10(B). Romera et al. [38] have studied the surface roughness of cassava starch cellulose blend films and reported that root means square roughness values of 29–45 nm. The result of surface roughness shown that the roughness of composite bioplastic is higher than starch bioplastic this increased rate of roughness may be due to the addition of TiO_2_.

### 4.7 Soil burial biodegradation test

**Fig. 11:**
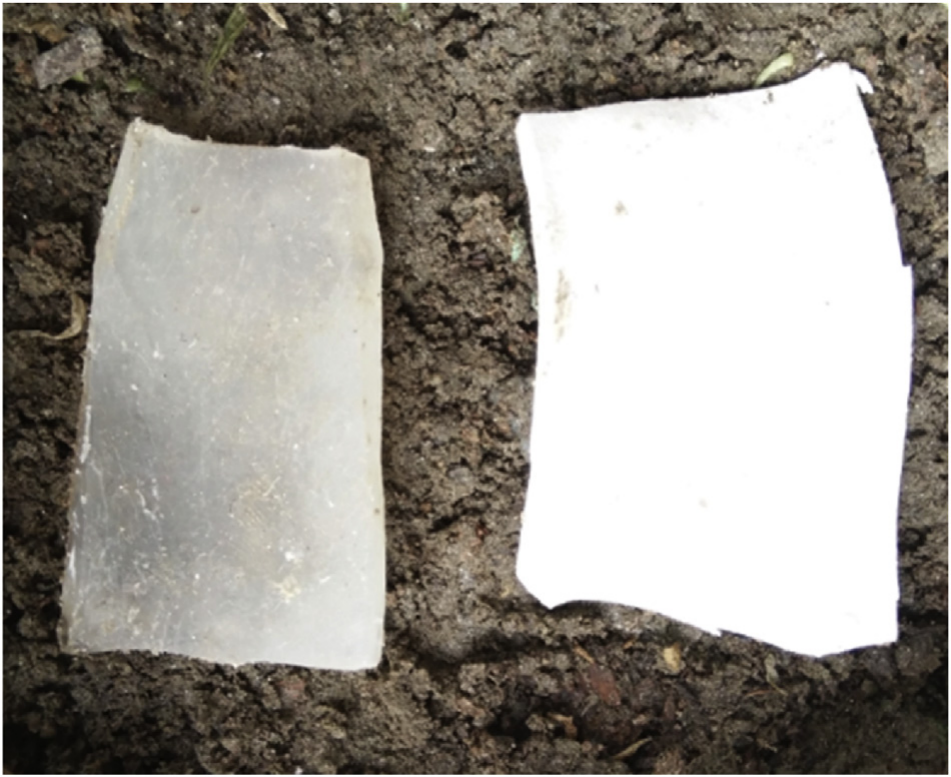
Physical Appearance of starch bioplastic and composite bioplastic samples before burial.

**Fig. 12:**
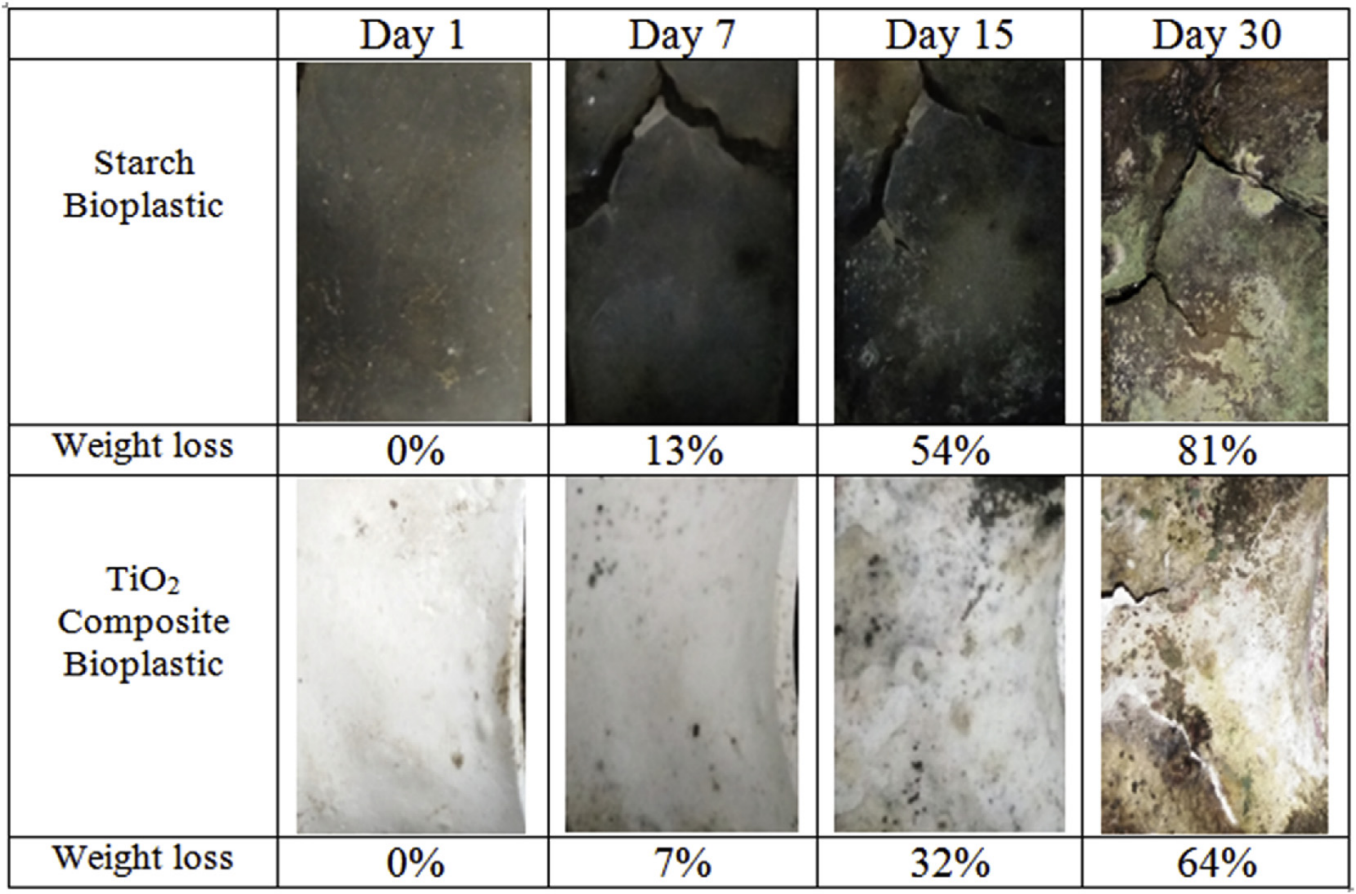
Degradation of starch bioplastic and composite bioplastic.

The degradation rate of the soil burial test was calculated from weight loss of the sample over time (Fig. 11). A direct way to measure the biodegradability of polymers is weight loss. The weight loss percentage of starch bioplastic and composite bioplastic are shown in Fig. 12. It is investigated from the figure that percent weight loss of both the samples increases continuously with increasing the number of days. Maximum % wt. loss 81% and 64% of starch bioplastic and composite bioplastic sample respectively were observed after one month; it's indicating that the samples continuously degrade with increase in the length of time. Tunma et al. [39] have noticed a similar trend in starch-based films, including with and without nanoparticles, which decomposed absolutely within 14 days. This result indicates the percentage of weight loss of starch bioplastic faster than composite bioplastic because microorganisms don't easily attack TiO_2_ composite bioplastic. The European standard EN13432 [40] requires that biodegradable plastics have 90% of their mass is fragmented in water, CO_2_, and biomass after six months. By the results analysis, both plastics were degraded quickly and can be considered biodegradable materials.

### 4.8 Comparative analysis

Table 6 shows the comparative analysis of composite bioplastic with starch bioplastic. The composite bioplastic has better physicochemical and thermal properties than starch bioplastic. Table 7 and Table 8 show a comparative analysis of starch bioplastics and composite bioplastic with relevant research. This analysis indicates that both bioplastics have improved their physicochemical and thermal properties.

**Table 6:** Comparative analysis of composite bioplastic with starch bioplastic.

Si. No.	Test/Analysis	Starch Bioplastic	Composite Bioplastic	Results Deviation	Remarks
01	Thermal decomposition (50% of weight loss)	291 °C	303 °C	12 °C	Increased (4.12%)
02	Melting temperature (T_m_)	297 °C	303 °C	6 °C	Increased (2.02%)
03	Glass Transition temperature (T_g_)	57.2 °C	66.8 °C	9.6 °C	Increased (16.78%)
04	Tensile Strength	3.55 ± 0.035 MPa	3.95 ± 0.039 MPa	0.4 ± 0.004 MPa	Increased (11.26 %)
05	Elongation (%)	88.1	62.5	- 25.6	Decreased (29.05 %)
06	Surface Roughness (Average)	0.6 μm	1 μm	0.4 μm	Moderate Decreased

**Table 7:** Comparative analysis of starch bioplastics with relative research.

Si. No.	Test/Analysis	Results	Available results		Results Deviation	Remarks
01	Thermal decomposition (50% of weight loss)	291 °C	250 °C	Nurul et al. [28]	41 °C	Increased (16.4%)
02	Melting temperature (T_m_)	297 °C	132.3 °C	Seyed et al. [27].	164.8 °C	Increased
03	Glass Transition temperature (T_g_)	57.2 °C	21.6 °C	Seyed et al. [27].	35.6 °C	Increased
04	Tensile Strength	3.55 MPa	2.66 MPa	Seyed et al. [27].	0.89 MPa	Increased (33.45 %)
05	Elongation	88.1	86.7	Seyed et al. [27].	1.4	Increased (1.6 %)
06	Surface Roughness (Average)	0.6 μm	26 nm	Romera et al. [38]		Comparative better

**Table 8:** Comparative analysis of composite bioplastics with relative research.

Si. No.	Test/Analysis	Results	Available results		Results Deviation	Remarks
01	Thermal decomposition (50% of weight loss)	303 °C	310 °C	Nurul et al. [28]	-7 °C	Decreased (2.25%)
02	Melting temperature (T_m_)	303 °C	136.6 °C	Seyed et al. [27].	166.4 °C	Increased
03	Glass Transition temperature (T_g_)	66.8 °C	35.3 °C	Seyed et al. [27].	35.6 °C	Increased
04	Tensile Strength	3.95 MPa	3.56 MPa	Seyed et al. [27].	0.39 MPa	Increased (10.95 %)
05	Elongation	62.5	62.79	Seyed et al. [27].	-0.29	Decreased (0.46 %)
06	Surface Roughness (Average)	1 μm	26 nm	Romera et al. [38]		Comparative better

## 5 Conclusions

In this study, it can be ascertained that starch-based bioplastics and composite bioplastic have been successfully characterizied by various analysis. The composite bioplastic is stronger than starch bioplastic with increased tensile strength and reduced elongation. From FTIR analysis the absorption band of the C–H and C–O–H at 2932 cm^−1^ and 1152.92 cm^−1^ for the starch bioplastic shifted to lower Wavenumbers at 2926.31 cm^−1^ and 1151.54 cm^−1^, which is confirmed the electrostatic interaction between the starch chains and titanium dioxide. SEM images show that Composite bioplastics have a better consistent surface in the compared to starch bioplastics for less void, hole and crack. DSC curves revealed that the Melting temperature (T_m_) and glass transition temperature (T_g_) of composite bioplastic showed a tendency to a higher than starch bioplastic, Which can be favorable for the packaging system. TGA indicates that the composite bioplastics have higher heat stability than the starch bioplastics. Soil burial biodegradation test indicates the biodegradability of starch bioplastic is more than composite bioplastic and also both plastics are highly biodegradable. By adding TiO_2_ to starch bioplastics, its properties have been improved. The preparation of starch bioplastic and composites bioplastic with better thermal, mechanical and chemical properties is a significant achievement. These products can be a appropriate alternative for the existing conventional plastics for its high biodegradable properties with suitable thermal and mechanical properties. Also starch is a renewable resource, cheap and easy to modify. Titanium dioxide nanoparticles have antimicrobial properties and composite bioplastics can be considered suitable for the food and pharmaceutical industry considering the experimental results. It will reduce reliance on petroleum polymers and environmental problems like today will not be intense.

## Declarations

### Author contribution statement

Md. Ruhul Amin: Conceived and designed the experiments; Wrote the paper.

Asaduzzaman Chowdhur: Contributed reagents, materials, analysis tools or data.

Md. Arefin Kowser: Performed the experiments.

### Funding statement

This research did not receive any specific grant from funding agencies in the public, commercial, or not-for-profit sectors.

### Competing interest statement

The authors declare no conflict of interest.

### Additional information

No additional information is available for this paper.
